# Respiratory Failure during BIS-Guided Sedation in a Patient with Relapsing Polychondritis: A Case Report

**DOI:** 10.3390/medicina59010065

**Published:** 2022-12-28

**Authors:** Jaesang Lee, Hosik Moon, Sungjin Hong, Jinyoung Chon, Hyejin Kwon, Hunwoo Park, Jiyung Lee

**Affiliations:** Department of Anesthesiology and Pain Medicine, College of Medicine, The Catholic University of Korea, Seoul 06591, Republic of Korea

**Keywords:** airway, bispectral index, relapsing polychondritis, respiratory failure, sedation, spinal anesthesia

## Abstract

Relapsing polychondritis (RP) is a rare autoimmune disorder that causes inflammation and deterioration of cartilaginous structures such as the ears, nose, joints and laryngotracheobronchial tree. A 42-year-old man receiving treatment for RP underwent open reduction and internal fixation of a femur fracture under spinal anesthesia and with sedation by propofol and remifentanil. The level of sedation was monitored via a bispectral index (BIS), and maintained at between 60 and 80. At the end of the operation, he lost consciousness and displayed weak respiratory effort. During mask ventilation, the patient was judged to have respiratory failure due to high end-tidal CO_2_ (EtCO_2_) concentration and respiratory acidosis in an arterial-blood-gas analysis (ABGA). Ventilation through a properly inserted laryngeal-mask-airway or endotracheal intubation were impossible; instead, a surgical tracheotomy was performed. After recovering from respiratory failure with ventilatory support in the intensive care unit (ICU), he experienced the same symptoms three more times, requiring ventilatory support. He was discharged with bilevel positive-airway-pressure (BiPAP), after successful adaptation.

## 1. Introduction

Relapsing polychondritis (RP) is a rare multisystem disease which is also classed as an autoimmune disorder. It affects cartilaginous structures and other connective tissues throughout the body with recurrent (and sometimes disseminated) inflammation [1]. Laryngotracheobronchial involvement is observed in up to 50% of patients with RP, and is a major cause of morbidity and mortality [2]. Along with granulomatosis with polyangiitis (GPA), RP is one of the main inflammatory diseases presenting with subglottic stenosis and tracheal stenosis. Compared to subglottic and circumferential GPA-related stenoses, RP-related stenoses are mostly tracheal, calcified, and strictly anterior [3]. Tracheal or bronchial narrowing and dynamic airway obstruction due to tracheobrochomalacia (TBM) are often attributed to airway obstruction [4]. It is critical that physicians and anesthesiologists avoid airway obstruction when sedating and anesthetizing a patient with RP. Knowledge and understanding of the pathophysiology of airway obstruction in RP is crucial.

Bispectral index (BIS) monitoring is widely used in clinical anesthesia to monitor the electrical activity of the cerebral cortex and the sedative ingredients of anesthesia. BIS monitoring reduces the risk of intraoperative awareness in surgical patients at high risk of awareness, and is useful for maintaining a constant depth of anesthesia during sedation [5,6]. Although there are conflicting reports about the relationship between the BIS value and the patient’s level of consciousness [7,8], it has been suggested that BIS monitoring may be useful for the objective early-detection of hypercapnic encephalopathy with depression of the level of consciousness [9]. In this case report with literature reviews, we describe the hypercapnic respiratory failure associated with decreased level of consciousness during sedation in a patient with RP, and the interpretation of the BIS value reflecting level of consciousness and depth of sedation.

## 2. Case Presentation

**Figure 1:**
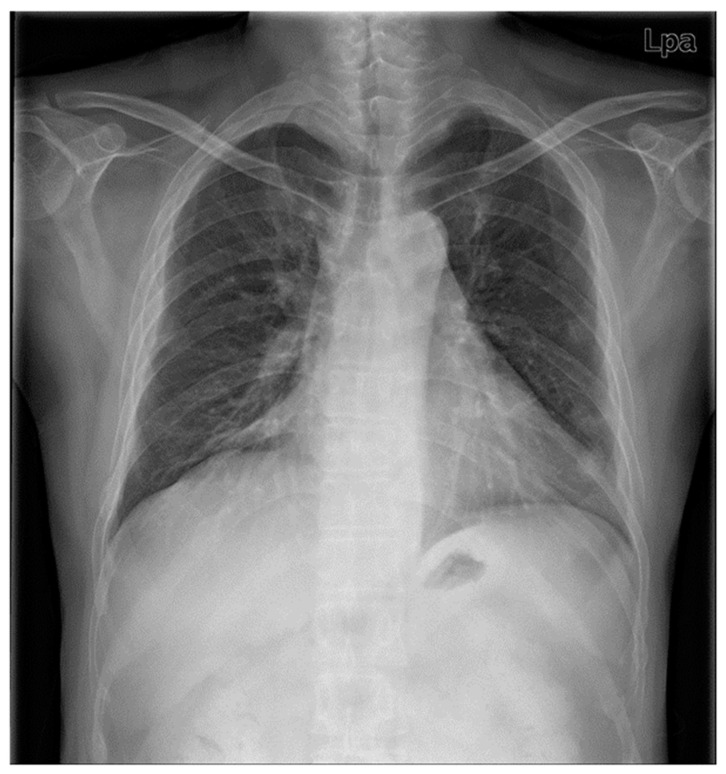
Preoperative chest radiography shows diffuse peribronchial cuffing in both lungs, with marked narrowing trachea, suggesting known relapsing polychondrits, and healed fracture at Rt. 8,9th and Lt. 8th rib.

**Figure 2:**
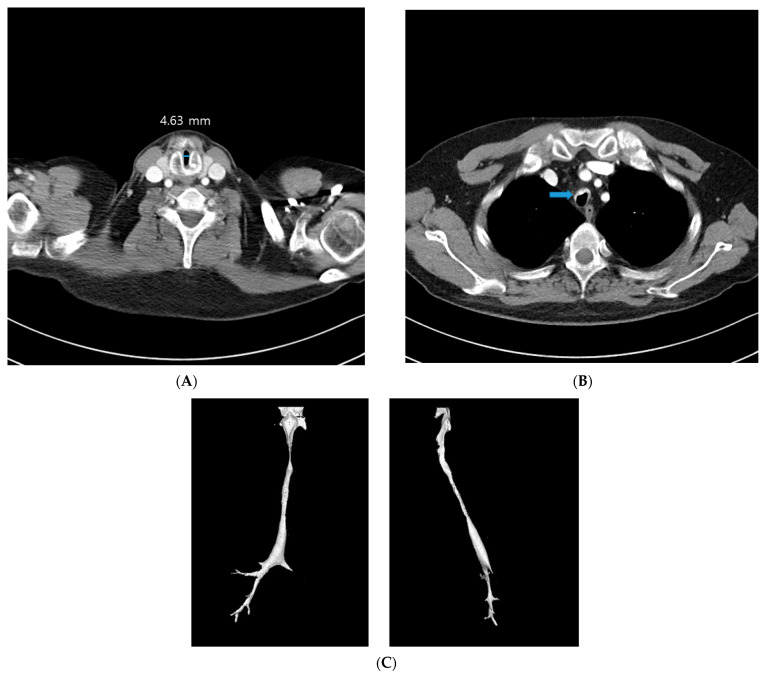
(**A**) CT shows subglottic stenosis at cricoid level with short-axis diameter 4.63 mm. (**B**) Calcification and anterolateral wall thickening of tracheobronchial tree (arrow) and resulting luminal narrowing. (**C**) Tracheobronchial tree shows irregular narrowing of the lumen and marked diffuse luminal-narrowing of left mainstem bronchus.

A 42-year-old male patient underwent an emergency operation, due to a left femur fracture. He had been diagnosed as RP 13 years prior to this, and continued to receive treatment. He lost his right-side hearing in childhood and subsequently lost his left-side hearing 13 years ago. His chest X-ray film showed diffuse peribronchial cuffing in both lungs, with marked narrowing of the trachea, suggesting RP (Figure 1). His previous CT revealed a subglottic stenosis with a short axis of 4.63 mm and a long axis of 13.9 mm (Figure 2A), anterolateral wall thickening, and calcification of the tracheobronchial tree, resulting in luminal narrowing (Figure 2B); this deformity was observed throughout the trachea (Figure 2C). His medication regime included prednisolone, hydroxychloroquine, celecoxib, and cyclosporine. He was also receiving treatment for bipolar disorder in the form of individual psychotherapy, aripiprazole, and escitalopram. The patient’s ASA (American Society of Anesthesiologists) physical status was Class 3.

When the patient entered the operating suite, the initial vital signs were blood pressure (BP) 136/86 mmHg, heart rate (HR) 75 beats per minute (bpm), and arterial oxygen saturation by pulse oximetry (SpO_2_) 93%. Spinal anesthesia was performed atraumatically in the right-lateral decubitus position, using a midline approach with a 25G spinal needle at L4–5 intervertebral space. We injected 12 mg of hyperbaric bupivacaine intrathecally. After the spinal block, the vital signs were stable: BP 132/76 mmHg, HR 78 bpm, and SpO_2_ 98%, with an oxygen supply of 3 L/min via a nasal cannula. The level of spinal block was checked by a visual analogue scale using a pinprick because of his deafness, and it seemed to be blocked to T10 dermatome.

Because the patient suffered from anxiety and insomnia, we infused 2% propofol (target plasma concentration, 2–2.5 µg/mL) and remifentanil (effect-site concentration 2 ng/mL) using a target-controlled infusion pump (Orchestra^Ⓡ^ Base Primea, Fresenius Kabi, France) under the monitoring of BIS, within a range of between 60 and 80. During sedation, we monitored end-tidal CO_2_ concentration (EtCO_2_) through a nasal cannula tip into the nares to monitor his respiratory rate, even though this did not provide an exact EtCO_2_ value. The operation started 35 min after the spinal block and over an hour later. Towards the end of the operation, we stopped the propofol and remifentanil infusion. However, when we attempted to rouse the patient after surgery using verbal and tactile stimuli, he did not respond. Although use of the drug ended with satisfactory speed, his respiration deteriorated rapidly until his SpO_2_ had decreased to 75%.

We started ventilatory support by mask and anesthesia workstation. The patient became hypertensive (BP 167/88 mmHg), tachycardiac (HR 128 bpm), remained unresponsive, and had very weak and sparse respiratory effort. We inserted a laryngeal mask airway (size 4 i-gel device), but ventilation proved very difficult. Next, tracheal intubations with an endotracheal tube of internal diameter (ID) 6.0 mm, and 5.0 mm were attempted, sequentially. The trials were conducted using a video laryngoscope, after the injection of 60 mg of succinylcholine. The glottic view remained intact, but the endotracheal tubes did not pass through the subglottic area. We injected 500 mg of methylprednisolone for the prevention of airway edema. The patient’s vital signs were: BP from 155/74 to 202/110 mmHg with the intermittent injection of nicardipine, HR from 128–145 bpm, EtCO_2_ 60–80 mmHg, and SpO_2_ 88–95%. The arterial-blood-gas analysis (ABGA) showed hypercapnic respiratory failure (pH 7.12, PaCO_2_ 86.9 mmHg, PaO_2_ 112.1 mmHg, HCO_3_^−^ 27.5 mmol/L). After a prompt decision to perform a tracheostomy, the otolaryngology team arrived, in anticipation of technical difficulties. The tracheotomy was carried out under local anesthesia with ventilatory support, while the patient was still obtunded. Through the open stoma of the third trachea ring, an ID 6.0 mm tracheotomy tube was inserted successfully, and was connected to the anesthesia workstation. The whole process, from recognizing respiratory failure to completion of tracheotomy, took approximately 50 min. The patient was then transported to the intensive care unit (ICU) for ventilator care. He stayed in the ICU for one week, for ventilator care.

On the eighth day, after successful weaning from the ventilator, the patient complained of dyspnea and became obtunded. His SpO_2_ was 88–95%. After oxygen therapy via a face mask, his ABGA showed severe respiratory acidosis (PH 7.14, PaCO_2_ 83.1 mmHg, PaO_2_ 113.4 mmHg, HCO_3_^−^ 27.7 mmol/L). He was readmitted to the ICU for ventilatory support once again. For long-term care, tracheal dilation and an exchange of the tracheotomy tube for a T-tube were planned by the ENT department.

The second operation was carried out under total intravenous anesthesia with remifentanil, propofol, and rocuronium bromide. After completion of endoscopic tracheal dilation and T-tube insertion, we stopped the infusion of anesthetics and injected sugammadex 200 mg. The patient’s recovery was complete, and he did not require postoperative ventilator care.

Unfortunately, the patient experienced two more bouts of hypercapnic respiratory failure during admission. After the physician applied a bilevel-positive-airway-pressure (BiPAP) ventilator during sleep, no more CO_2_ retention occurred. The patient was discharged with BiPAP 1 month after T-tube insertion. He provided written informed consent for the publication of this report.

## 3. Discussion and Conclusions

Because RP is a rare autoimmune disorder, with an incidence of 3.4–4.5 cases per million per year [2], it is also uncommon to see these patients for anesthesia. Sometimes, regional anesthesia is used to avoid difficulties surrounding airway management [10]. When general anesthesia is necessary, cautious airway management is needed [11], or an oxygen supply should be guaranteed by extracorporeal membrane oxygenation [12]. During general anesthesia, respiratory insufficiency associated with dynamic hyperinflation or difficult extubation have been reported [13,14]. Respiratory problems are the main concern in the anesthetic management of RP patients. In respiratory monitoring during sedation, in this case the EtCO_2_ was not an accurate indicator of ventilation because it was measured through nasal cannula. Its role was as an index to provide the anesthesiologist with an evaluation of the respiratory rate and pattern. The hypercapnia detected after the mask ventilation indicated that respiratory insufficiency had already progressed, but it was not detected by EtCO_2_ monitoring. Therefore, during sedation in patients with possible hypercapnia, the exact monitoring of EtCO_2_ through the use of a tight mask or the arterial pressure of CO_2_ by ABGA, would be helpful.

In severe exacerbations of chronic obstructive pulmonary disease (COPD), hypercapnia leads to respiratory acidosis and neurological abnormalities, including neurological–cognitive alterations and a reduction in the level of consciousness, which is known as hypercapnic encephalopathy. In patients with hypercapnic encephalopathy, BIS seems to adequately reflect the state of consciousness [9]. In this case, the BIS value appeared to illustrate the decreased level of consciousness from hypercapnia, in addition to the depth of sedation.

The failure of endotracheal intubation, due to the characteristics of the RP in this case, was predicted prior to anesthesia. The mechanisms responsible for airway obstruction in RP are inflammatory airway edema in the active stage, dynamic collapse of the airway which is secondary to cartilage destruction, and contraction and airway stenosis by fibrous-tissue formation. Radiologically, anterior-airway wall thickening, with or without calcification, and with sparing of the posterior membranous wall, is the most common and pathognomonic finding. Airway stenosis and TBM are also highly prevalent. The most frequent finding during bronchoscopy was TBM, subglottic stenosis, followed by focal stenosis in the tracheobronchial tree [15]. During bronchoscopy, the use of conscious sedation may depress ventilation and relax the respiratory muscles, so that a relatively stable airway becomes unstable [16]. The obstruction worsens when supine, presumably due to airway collapse [17]. Any causes that induce a dyspneic sensation and further respiratory effort, exacerbate dynamic-airway obstruction by increasing positive intrathoracic pressure. In this patient, all these factors—the existing dynamic collapse of the airway and fixed airway-stenosis; the depression of ventilation and relaxation of the respiratory muscles by conscious sedation; and worsening obstruction, due to his supine position—contributed to respiratory failure.

Considering the potential causative factors of frequent respiratory failure in this patient, the most probable contributor is sleep, including sedation. It was clear that one of the three respiratory-failure symptoms in the ward was related to sleep and fever. In 43% of patients with advanced COPD, chronic daytime hypercapnia may be preceded by sleep hypoventilation, with nocturnal hypercapnia [18]. REM-related sleep hypoventilation caused by diminished activities of the intercostal and accessory respiratory muscles and dampened hypoxic and hypercapnic ventilatory-responses could provoke a number of respiratory and non-respiratory symptoms, as well as overt hypercapnic-respiratory-failure [19].

We presented a case report of respiratory failure during BIS-guided sedation in a patient with relapsing polychondritis, considering the occurrence and mechanisms. Fixed-airway stenosis and the dynamic collapse of the airway seen in RP, as well as sedative drugs inducing respiratory depression and the relaxation of the respiratory muscles, seemed to contribute to respiratory failure. While monitoring a patient with RP during sedation, the BIS value requires careful interpretation, because it can reflect not only the degree of drug-induced sedation, but also the changes in the level of consciousness caused by other factors, such as hypercapnia.

## Data Availability

Data sharing is not applicable to this article, as no datasets were generated or analyzed during the current study.
